# Association of procalcitonin levels with the progression and prognosis of hospitalized patients with COVID-19

**DOI:** 10.7150/ijms.48396

**Published:** 2020-09-09

**Authors:** Ze-Ming Liu, Jin-Peng Li, Shi-Pei Wang, Dan-Yang Chen, Wen Zeng, Si-Chao Chen, Yi-Hui Huang, Jiang-Long Huang, Wei Long, Man Li, Rong-Fen Gao, Liang Guo, Xiao-Hui Wu

**Affiliations:** 1 Department of Plastic Surgery, Zhongnan Hospital of Wuhan University, Wuhan, 430071, China.; 2 Department of Thyroid and Breast Surgery, Zhongnan Hospital of Wuhan University, Wuhan, 430071, China.; 3 Department of Ophthalmology, Zhongnan Hospital of Wuhan University, Wuhan, 430071, China.; 4 Department of Rheumatology and Immunology, Tongji Hospital, Tongji Medical College, Huazhong University of Science and Technology, Wuhan, Hubei, China.; 5 Department of Neurosurgery, Zhongnan Hospital of Wuhan University, Wuhan, 430071, China.

**Keywords:** COVID-19, risk factor, procalcitonin, prognosis, disease progression

## Abstract

**Rationale:** Coronavirus disease 2019 (COVID-19) was first announced in Wuhan, and has rapidly evolved into a pandemic. However, the risk factors associated with the severity and mortality of COVID-19 are yet to be described in detail.

**Methods:** We retrospectively reviewed the information of 1525 cases from the Leishenshan Hospital in Wuhan. Univariate and multivariate Cox regression analyses were generated to explore the relationship between procalcitonin (PCT) level and the progression and prognosis of COVID-19. Univariate and multivariate logistic regression analyses were performed to explore the relationship between disease severity in hospitalized patients and their PCT levels. Survival curves and the cumulative hazard function for COVID-19 progression were conducted in the two groups. To further detect the relationship between the computed tomography score and survival days, curve-fitting analyses were performed.

**Results:** Patients in the elevated PCT group had a higher incidence of severe and critical severity conditions (*P <* 0.001), death, and higher computed tomography (CT) scores. There was an association between elevated PCT levels and mortality in the univariate ((hazard ratio [1], 3.377; 95% confidence interval [2], 1.012-10.344; *P =* 0.033) and multivariate Cox regression analysis (HR, 4.933; 95% CI, 1.170-20.788; *P =* 0.030). Similarly, patients with elevated PCT were more likely to have critically severe disease conditions in the univariate (odds ratio [2], 7.247; 95% CI, 3.559-14.757; *P <* 0.001) and multivariate logistic regression analysis (OR, 10.679; 95% CI, 4.562-25.000; *P <* 0.001). Kaplan-Meier curves showed poorer prognosis for patients with elevated PCT (*P =* 0.024). The CT score 1 for patients with elevated PCT peaked at day 40 following the onset of symptoms then decreased gradually, while their total CT score was relatively stable.

**Conclusion:** PCT level was shown as an independent risk factor of in-hospital mortality among COVID-19 patients. Compared with inpatients with normal PCT levels, inpatients with elevated PCT levels had a higher risk for overall mortality and critically severe disease. These findings may provide guidance for improving the prognosis of patients with critically severe COVID-19.

## Introduction

In December 2019, unknown epidemic information was found in a seafood market in Wuhan City, Hubei Province, China 3. Subsequently, this pneumonia became a pandemic throughout China, with sporadic cases reported globally and involving all aspects of daily life 4. On 11 February 2020, World Health Organization (WHO) named the novel coronavirus disease as COVID-19 5. As of May 5, 2020, a total of 3,517,345 people worldwide have been diagnosed with COVID-19, and 243,401 deaths have been reported 6. Owing to the rapid increase in international cases, the WHO has increased the transmission and impact risk assessment score of COVID-19 to a very high risk at the global level 7. The most common symptoms of COVID-19 are nonspecific, including fever, cough, dyspnea, shortness of breath, and fatigue 8. According to the assessment, the causative pathogen is the severe acute respiratory syndrome coronavirus 2 (SARS-CoV-2). The main clinical manifestations are pneumonia with acute respiratory distress syndrome, septic shock and other complications 9. Although the incidence rate of COVID-19 is so high, its mortality is not as terrible as it is expected. Only a small proportion (5%) may become a critical illness 10 and eventually lead to death. However, even though most cases are not serious, this disease is still distressing and can be long-lasting. Data from eight hospitals in Georgia shows that among patients without high-risk conditions, 22.5% were admitted to the ICU, and 5.1% died while in the hospital 11.

At the beginning of the outbreak, most of the cases had a history of travel to China or Wuhan or contact with Chinese people. Later, COVID-19 is believed to spread via close contact by coughing and sneezing, aerosols and respiratory droplets, and even eye secretions, saliva, urine, and stools 12. Inflammatory responses play a key role in the pathology of COVID-19, and increases in inflammatory cytokine levels will increase the severity of this disease 13. The fifth edition of “Diagnosis and Treatment of COVID-19” suggests monitoring the level of cytokines to improve the treatment effect and reduce mortality 14. One study has shown that although the total number of patients with COVID-19 presenting increased procalcitonin (PCT) levels appears to be limited, continuous measurement of PCT levels may play a role in predicting the progression of this disease into the severe forms 15. In general, PCT levels are high following bacterial infection, but relatively low after viral infection. A previous finding that PCT level was an independent factor for predicting the severity of COVID-19 is not accurate 16. Thus, further investigations regarding the association between PCT levels and the severity of COVID-19 are needed.

In this retrospective study, we collected clinical data of 1525 cases in Leishenshan Hospital, Wuhan, Hubei Province, China, which is a temporary hospital specialized and designated for the treatment of patients with COVID-19, from February 8, 2020 to April 15, 2020. We aimed to describe the characteristics of COVID-19 and investigate the relationship between PCT levels and the progression and prognosis of COVID-19 among hospitalized patients.

## Methods

### Study design and participants

A cohort of 1880 patients who were laboratory-confirmed to have COVID-19 between February 9 and March 18 at the Leishenshan Hospital were enrolled in this retrospective study. The final date of follow-up was April 15, 2020. The information of these patients was obtained from medical records, and the data were reviewed by two physicians independently. Patients with unclear PCT levels were excluded from the study. A total of 1525 patients were included in the study after exclusion. Patient information, including demographic characteristics, history of comorbidity, symptoms, laboratory findings, computed tomography (CT) images, and treatment, was extracted from the original medical records. This study was approved by the Research Ethics Commission of Zhongnan Hospital of Wuhan University (approval number: 2020074). Patient consent was waived by the ethics committee because this infectious disease was rapidly evolving.

### The primary outcomes in this study

The primary outcomes of this study were patient survival (alive or dead) and the worst disease severity during hospitalization. The patients were divided into two groups according to the results of PCT, normal PCT group (PCT<0.05 ng/ml) and elevated PCT group (PCT≥0.05 ng/ml). Disease severity was classified as general, mild, severe, and critical based on the seventh edition of the “Diagnosis and Treatment of COVID-19” published by the Chinese National Health Commission. In-hospital stays and CT findings were the other two important outcomes of our study. All CT images were independently interpreted by two experienced radiologists; findings from inconsistent images were finalized through discussion. On the basis of previous studies and the CT image characteristics of COVID-19, we used an optimized semi-quantitative scoring system to assess pulmonary inflammation. Score 1 was calculated based on the number of categories of findings identified on the pulmonary CT images among the following: ground-glass opacities (GGOs), reticulation or cord change, consolidation, and pleural effusions. One point was awarded for each category identified, and score 1 was calculated as the sum of all points. Score 2 was calculated based on the area of involvement in the lung lobes: no involvement, 0 point; < 25% involvement, 1 point; 26-50% involvement, 2 points; 51-75% involvement, 3 points; and 76-100% involvement, 4 points. The total CT score was the sum of score 1 and score 2.

### Statistical analysis

Continuous variables were expressed as the median and interquartile range (IQR), and categorical variables were expressed as number and percentage (%). Independent group t-test or Mann-Whitney test were used for detecting differences in the mean values of continuous variables between the normal PCT group and the elevated PCT group. With regard to the proportions of categorical variables, comparisons between these two groups were performed by using the χ^2^ test or Fisher exact test when the data were limited. Univariate and multivariate Cox regression analyses were generated to elucidate the relationship between PCT level and the prognosis of COVID-19 patients. We used logistic regression analysis to assess the risk between PCT level and critical illness. Variables enrolled in the multivariate analysis included age, history of cardiovascular diseases, white blood cell (WBC) count, platelet (PLT) count, lymphocyte count, and D-dimer levels. Patient survival curves and a cumulative hazard function for COVID-19 progression in both groups were analyzed using Kaplan-Meier analysis, along with the log-rank test. To further explore the relationship between CT score and days of hospitalization, curve-fitting analysis was carried out. All statistical analyses were conducted using SPSS 23.0 (IBM Corporation, Armonk, NY, USA). Statistical significance was considered at two-sided P-values of less than 0.05.

## Results

### Demographic, clinical Information and laboratory findings

The characteristics of the subjects from the normal PCT group versus those of subjects from the elevated PCT group on admission are provided in **Table 1.** Among the 1525 participants with clear PCT data, 1008 were designated to the normal PCT group (median age 57 [IQR 47-66] years; 60.6% women) and the remaining 517 were allocated to the elevated PCT group (median age 63 [IQR 53-71] years; 38.7% women). There were significant differences in age and gender between the two groups (*P <* 0.001). In both groups, patients with cardiovascular comorbidities were the most common (21.4% and 19.0% for elevated and normal PCT groups, respectively), followed by those with endocrine diseases (8.1% and 7.5%, respectively). Although there was no significant difference in comorbidity between the two groups, the incidence of pulmonary diseases (1.9%) in the elevated PCT group was significantly lower than that in the normal PCT group (6.3%). In contrast, the incidence of neurological diseases showed an opposite trend (4.8% vs 2.1%; *P =* 0.003). The most common complaints presented by patients in the two groups were respiratory symptoms (79.9% vs 84.6%, respectively).

There were numerous differences in laboratory findings between the elevated PCT group and the normal PCT group (**Table 2**), including higher lymphocyte and monocyte counts, lower platelet count, as well as higher levels of albumin and creatine in the elevated PCT group. There were significant differences between the two groups in each index of blood coagulation test (**Table 3**).

In terms of in-hospital treatment, the normal PCT group had a higher percentage of patients receiving antibiotic (34.5% vs. 18.0%; *P <* 0.001), antiviral (99.7% vs. 95.1%; *P <* 0.001), and antimalarial (90.8% vs. 40.0%; *P <* 0.001) drugs than the elevated PCT group (**Table 4**). There were significant differences between the two groups in terms of disease severity at admission and worst severity during hospitalization (all variables, *P <* 0.001). The median survival days in the normal and elevated PCT groups were 21 and 14 days, respectively. Compared with the normal PCT group, more patients had higher CT scores in the elevated PCT group (71.7%), and mortality was higher in the elevated PCT group (1.4%).

### Survival analysis

Univariate Cox regression analysis revealed that the PCT level was positively correlated with the risk of in-hospital death (hazard ratio 1, 3.377; 95% confidence interval [CI], 1.102-10.344; *P =* 0.033). After adjustment for age, history of cardiovascular diseases, WBC, PLT, and lymphocyte count, and D-dimer levels, the same trend was observed in the multivariate Cox regression analysis (HR, 4.933; 95% CI, 1.170-20.788; *P =* 0.030) (**Table 5**).

Univariate logistic regression analysis showed that PCT level (odds ratio [OR], 7.247; 95% CI, 3.559-14.757; *P <* 0.001) was positively correlated with the risk of in-hospital death. After adjustment for age, history of cardiovascular diseases, WBC, PLT, and lymphocyte count, and D-dimer levels, the same trend was observed in the multivariate Cox regression analysis (OR, 10.679; 95% CI, 4.562-25.000; *P <* 0.001) (**Table 6**).

**Figure 1:**
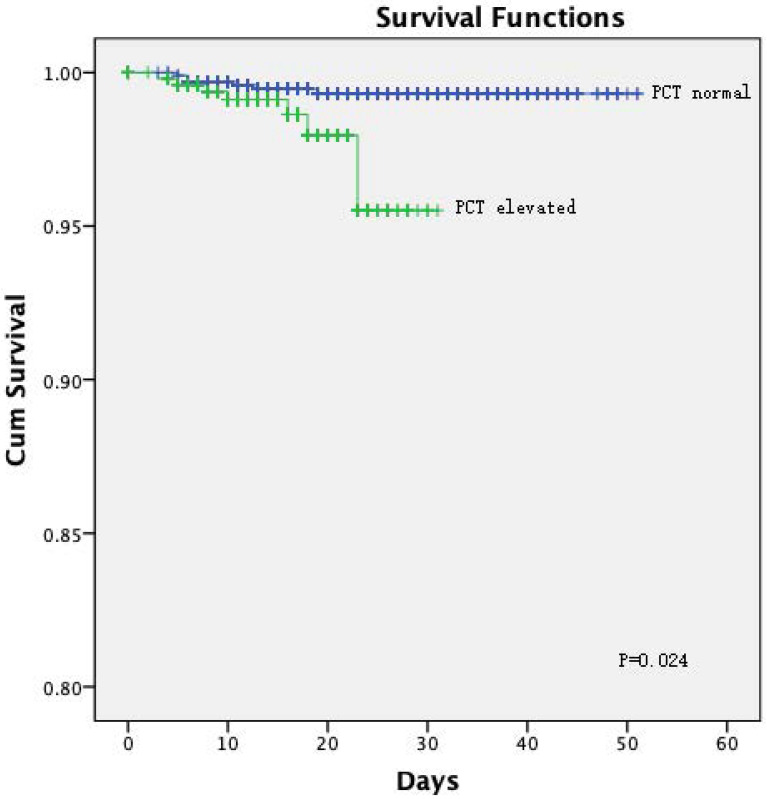
[missing legend].

**Figure 2:**
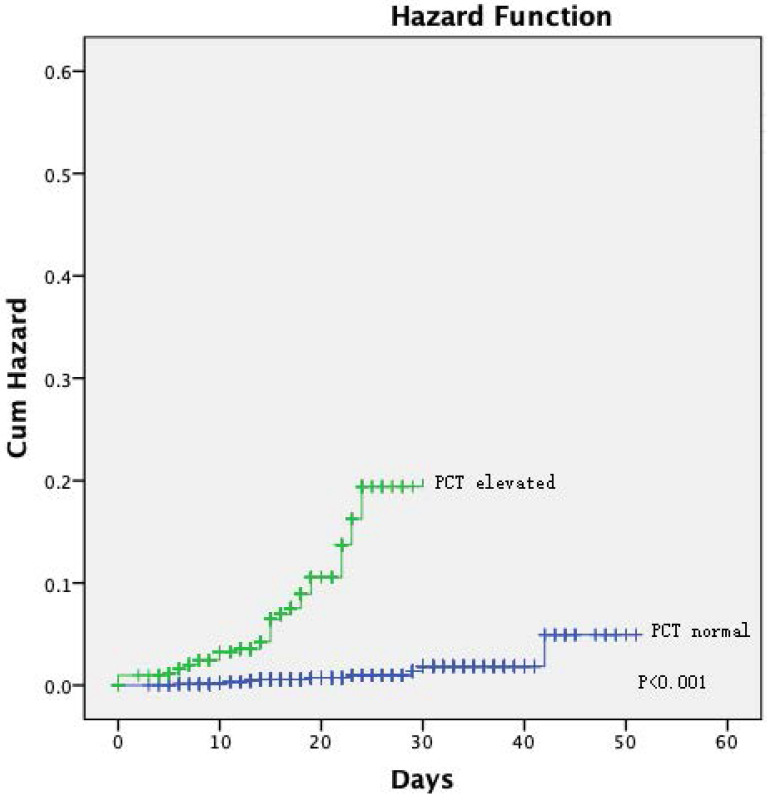
[missing legend].

The Kaplan-Meier curves showed a significantly better prognosis for patients in the normal PCT group than in the elevated PCT group (*P =* 0.024; **Figure 1**). The cumulative hazard function for COVID-19 progression was generated by applying the highest severity during hospitalization as the event. The result showed that the risk of developing severe disease was significantly higher in the elevated PCT group than in the normal PCT group (*P <* 0.001, **Figure 2**).

### Evaluation for chest CT images

**Figure 3:**
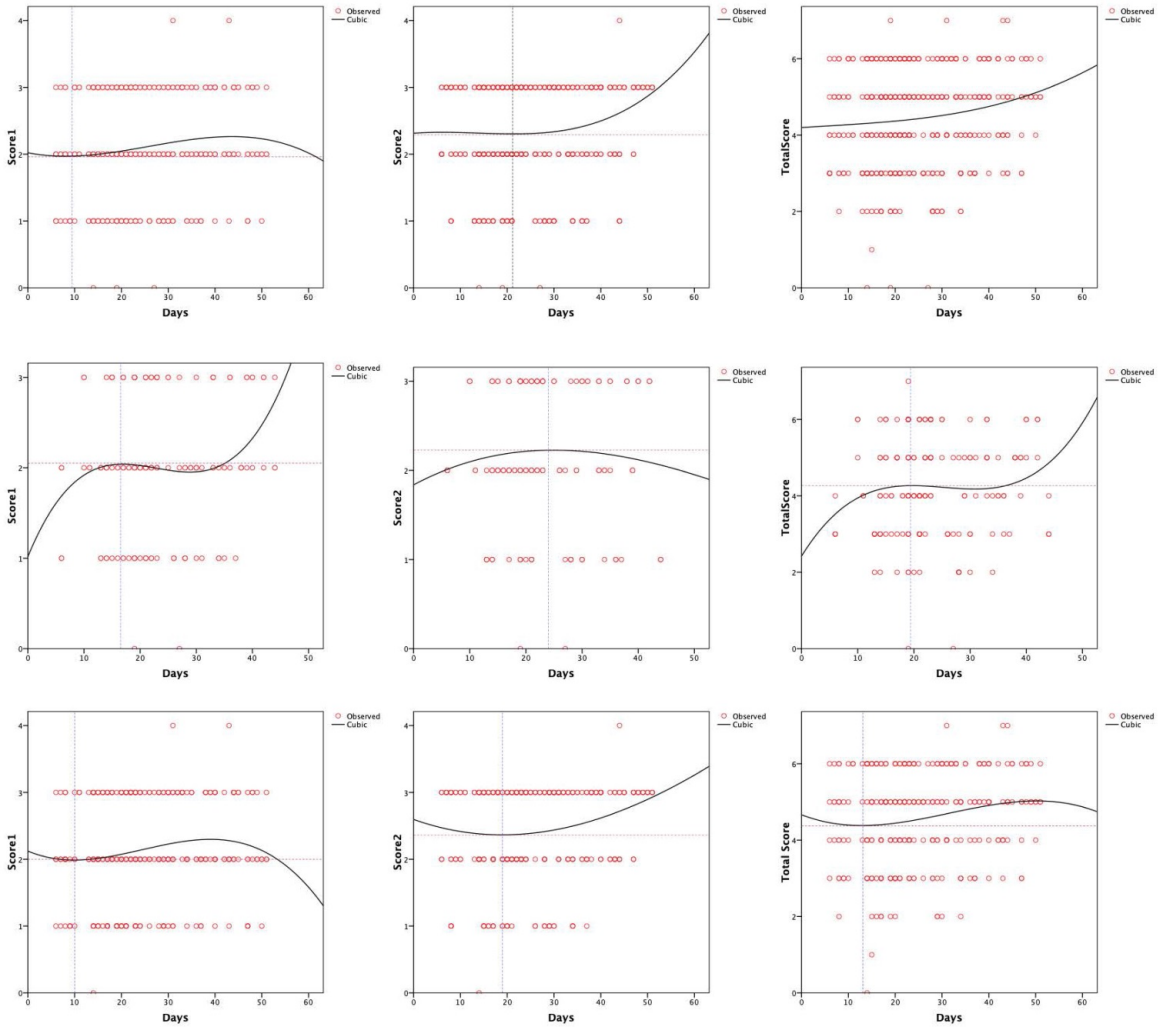
[missing legend].

The peak of score 1 for all the patients was 1.96 on day 9 (**Figure 3A**), 2.05 on day 17 in the normal PCT group (**Figure 3D**), and 2.0 on day 10 in the elevated PCT group (**Figure 3G**). For all patients, score 2 reached a trough of 2.29 at 20 days (**Figure 3B**). And in the elevated PCT group, score 2 was the lowest at 19 days, and then began to rise (**Figure 3H**). Score 2 for the normal PCT patients was 2.23 on day 24 (**Figure 3E**). The total CT score of all patients had increased to above 4 after symptoms appeared (**Figure 3C**). The normal PCT group reached a platform stage after the score reached 4.27 on day 19, and the score continued to increase 13 days later (**Figure 3F**). The fitting curve for imaging manifestations of the types of lung inflammation in the elevated PCT group showed a trend of first increasing, then decreasing (**Figure 3I**).

## Discussion

In this study, 1525 cases in the Leishenshan Hospital in Wuhan City were enrolled for the investigation of the effect of PCT level on the prognosis of patients with COVID-19. The elevated PCT group had a higher risk of critical disease than the normal PCT group (6.8% vs 1.0%, respectively). Cox proportional hazard model analysis showed that elevated PCT level was significantly associated with a higher risk of overall mortality among COVID-19 patients, even after adjustment for age, history of cardiovascular diseases, WBC, PLT, and lymphocyte count, and D-dimer levels as potential confounding factors. In addition, the risk of critical disease in the elevated PCT group was 3.559 times higher than that in the normal PCT group. This effect was even more significant after adjustment.

PCT is a glycoprotein without hormonal activity; it is the precursor of calcitonin. Previous clinical studies have shown that patients with viral pneumonia, including SARS pneumonia, had low PCT levels, i.e., less than 1 ng/ml; in contrast, PCT level is higher in bacterial and fungal pneumonia 17. Given that COVID-19 and SARS have similar clinical features 18, it is speculated whether PCT can also predict the prognosis of COVID-19. Patients in the intensive care unit (ICU) have a significantly higher level of GSCF, IP10, MCP1, and TNF-α than those non-ICU patients, suggesting that a cytokine storm might be an underlying cause of disease severity 19, 20. As a useful marker of systemic bacterial infection, PCT has higher specificity and sensitivity, compared with acute phase proteins such as C-reactive protein and interleukin-6 levels, even in a medical intensive care unit 21. Similar to our study, recent studies in patients with COVID-19 have also reported that the increase of PCT may be related to the severity of the disease 22, 23. These studies, as well as our results, suggest that bacterial infection may be present in many patients, especially in severe cases 8. However, it has never been systematically associated with virological research to assess its ability to identify deterioration 24. Further studies are needed to better understand the underlying biological mechanisms involved in the association between the PCT level and adverse outcomes in COVID-19.

The main initial symptoms of COVID-19 include fever, most of which are high fever in a few days, which cannot be relieved by conventional anti-infective drugs, cough, headache and muscle pain or fatigue 25. Our study showed that about 80% of patients presented with symptoms of fever or fatigue and respiratory symptoms. Both the normal and elevated PCT group had a similar rate of symptoms when admitted to the hospital such as fever, myalgia, respiratory system symptoms, digestive system symptoms, nervous system symptoms and other system symptoms. It should be emphasized that some asymptomatic persons are infected with SARS-CoV-2 26. One study has shown that nearly 80% of patients have normal or decreased white blood cell counts 25, which is similar to our results. The rate of leukocyte count increase in the elevated PCT group was slightly higher than that in the normal PCT group, although the difference was not statistically significant.

High-resolution CT allows the objective evaluation of lung lesions, thus enabling better understanding of the pathogenesis of the disease. With serial CT examinations, the occurrence, development, and prognosis of the disease can be better understood 27. Compared with RT-PCR analysis of sputum, throat swab, or lower respiratory tract specimens and the sequencing of viral genes, CT imaging can reveal typical features that contribute to the diagnosis of COVID-19, which can enable the rapid screening of patients and stratification of patients' severity, leading to the development of effective treatment strategies 28. The main CT manifestations in the early phase of COVID-19 included multifocal peripheral and basal GGOs, crazy paving patterns, traction bronchiectasis, and air bronchogram signs. A progressive transition to consolidation, together with pleural effusion, extensive small lung nodules, irregular interlobular or septal thickening, and adenopathy characterize the advanced phase of the disease 29. Researchers hold the opinion that the different nature and scope of lung tissue damage may be related to the duration of COVID-19 29. In our study, we analyzed the CT score trends of inpatients. In the normal PCT group, both score 1 and the total score increased gradually after the symptoms appeared (**Figure 3D and 3F**). However, there was no significant peak of increase in the elevated PCT group, and even the score 1 decreased again at approximately 40 days (**Figure 3G and 3I**). In addition, our study showed that the baseline score of the patients from the elevated PCT group was higher than that of the subjects from the normal PCT group. This may be due to the fact that patients with elevated PCT showed critical manifestations at the early stage of the disease. The condition of these patients was thus, better controlled because of the more advanced treatment at the early stage. This may indicate the response to relevant treatment measures. Further, whether empirical antibiotic coverage is needed in the case of a possible bacterial superinfection warrants further investigations.

Nevertheless, this study had several limitations. First, this was a retrospective study; thus, inpatient data, such as laboratory reports and past medical history, may be incomplete. These missing values may have contributed to research bias. Second, patients at the Leishenshan Hospital may have either self-medicated or received treatment at other isolation points prior to their hospital admission. In the absence of this information, our conclusions regarding the factors associated with prognosis may be unreliable. Third, because all subjects in our study resided in Wuhan, Hubei, China, these data need further validation for application in geographically diverse, prospective, cohort studies. If conditions permit, further study of the biological mechanism of PCT on the progress of COVID-19 will be performed.

## Conclusion

PCT level was shown to be an independent risk factor for in-hospital mortality among COVID-19 patients. Inpatients with elevated PCT levels had a higher risk of overall mortality than those with normal PCT levels. COVID-19 patients with elevated PCT levels were more likely to develop critically severe disease. Our findings may provide guidance for improving the prognosis of patients with critically severe COVID-19 infection.

## Figures and Tables

**Table 1:** Demographic characteristics and symptoms of 1525 patients with COVID-19

Covariates	All patients (n=1525), n (%)	Normal PCT (n=1008), n (%)	Elevated PCT (n=517), n (%)	*P* value
**Gender**				<0.001*
Female	810 (53.1)	610 (60.5)	200 (38.7)	
Male	715 (46.9)	398 (39.5)	317 (61.3)	
**Age, median (IQR)**	59 (49-68)	57 (47-66)	63 (53-71)	<0.001*
**Any Comorbidity**	472 (31.0)	322 (31.9)	150 (29.0)	0.241
Cardiovascular diseases	314 (20.6)	216 (21.4)	98 (19.0)	0.258
Pulmonary diseases	73 (4.8)	63 (6.3)	10 (1.9)	<0.001*
Endocrine diseases	121 (7.9)	82 (8.1)	39 (7.5)	0.686
Malignancy	51 (3.3)	40 (4.1)	11 (2.1)	0.058
Digest system diseases	39 (2.6)	28 (2.8)	11 (2.1)	0.446
Neurological diseases	46 (3.0)	21 (2.1)	25 (4.8)	0.003*
**Initial symptoms, n (%)**				
Fever or fatigue	554 (78.8)	300 (78.1)	254 (79.6)	0.628
Respiratory symptoms	577 (82.1)	307 (79.9)	270 (84.6)	0.106
Digestive symptoms	77 (11.0)	39 (10.2)	38 (11.9)	0.458
Neurological symptoms	24 (3.4)	11 (2.9)	13 (4.1)	0.379

Note: * represents *p* value <0.05.

**Table 2:** Laboratory test results of 1525 patients with COVID-19

Covariate	All patients (n=1525) Median (IQR) / n (%)	Normal PCT (n=1008) Median (IQR) / n (%)	Elevated PCT (n=517), Median (IQR) / n (%)	*P* value	Reference range
**Leucocyte count, × 10⁹/L**	5.68 (4.69-6.92)	5.63 (4.61-6.88)	5.8 (4.83-7.0)	0.188	3.5-9.5
3.5-9.5	1357 (89.1)	886 (88.0)	471 (91.3)	0.016*	
< 3.5	90 (5.9)	72 (7.1)	18 (3.5)		
> 9.5	76 (5.0)	49 (4.9)	27 (5.2)		
**Neutrophil count, × 10⁹/L**	3.28 (2.53-4.29)	3.28 (2.51-4.3)	3.29 (2.6-4.23)	0.994	1.8-6.3
1.8-6.3	1324 (86.9)	865 (85.9)	459 (89.0)	0.246	
< 1.8	101 (6.6)	72 (7.1)	29 (5.6)		
> 6.3	98 (6.4)	70 (7.0)	28 (5.4)		
**Lymphocyte count, × 10⁹/L**	1.59 (1.23-1.97)	1.57 (1.2-1.95)	1.62 (1.27-2.02)	0.048*	1.1-3.2
1.1-3.2	1239 (81.2)	818 (81.2)	421 (81.4)	0.944	
< 1.1	267 (17.5)	178 (17.7)	89 (17.2)		
> 3.2	19 (1.2)	12 (1.2)	7 (1.4)		
**Erythrocyte count, × 10^12^/L**	4.11 (3.76-4.47)	4.1 (3.76-4.43)	4.12 (3.75-4.53)	0.610	4.3-5.8
4.3-5.8	526 (34.5)	336 (33.4)	190 (36.8)	0.360	
< 4.3	987 (64.8)	665 (66.0)	322 (62.4)		
> 5.8	10 (0.7)	6 (0.6)	4 (0.8)		
**Monocyte count, × 10⁹/L**	0.5 (0.40-0.63)	0.5 (0.39-0.62)	0.52 (0.42-0.64)	0.042*	0.1-0.6
0.1-0.6	1073 (70.4)	725 (71.9)	348 (67.3)	0.124	
< 0.1	5 (0.3)	4 (0.4)	1 (0.2)		
> 0.6	447 (29.3)	279 (27.7)	168 (32.5)		
**Hemoglobin, g/L**	125 (114-136)	125 (115-135)	126 (113.5-137)	0.116	130.0-175.0
130.0-175.0	580 (38.1)	373 (37.0)	207 (40.1)	0.476	
< 130.0	938 (61.6)	631 (62.7)	307 (59.5)		
> 175.0	5 (0.3)	3 (0.3)	2 (0.4)		
**Platelet count, × 10⁹/L**	228 (185-278)	230 (185-282)	225 (183.5-272.5)	0.036*	125.0-350.0
125.0-350.0	1322 (86.7)	862 (85.5)	460 (89.0)	0.068	
< 125.0	70 (4.6)	46 (4.6)	24 (4.6)		
> 350.0	133 (8.7)	100 (9.9)	33 (6.4)		
**Albumin, g/L**	37.6 (34.8-39.9)	37 (34.4-39.5)	38.6 (36.25-40.9)	< 0.001*	40-55
40-55	372 (24.5)	199 (19.9)	173 (33.5)	< 0.001*	
<40	1144 (75.5)	801 (80.1)	343 (66.5)		
**Alanine aminotransferase, U/L**	22 (14-37)	21 (14-35)	25 (15-41)	0.065	9-50
9-50	1210 (79.8)	809 (80.9)	401 (77.7)	0.309	
<9	86 (5.7)	52 (5.2)	34 (6.6)		
>50	220 (14.5)	139 (13.9)	81 (15.7)		
**Aspartate aminotransferase, U/L**	20 (16-27)	19 (16-26)	21 (16-27.5)	0.799	15-40
15-40	1106 (73.0)	732 (73.2)	374 (72.5)	0.927	
< 15	271 (17.9)	176 (17.6)	95 (18.4)		
> 40	139 (9.2)	92 (9.2)	47 (9.1)		
**Total bilirubin, μmol/L**	9.1 (7-12.0)	8.9 (6.8-11.9)	9.5 (7.4-12.05)	0.094	5.0-21.0
5.0-21.0	1350 (89.1)	891 (89.1)	459 (89.0)	0.834	
< 5.0	107 (7.1)	72 (7.2)	35 (6.8)		
> 21.0	59 (3.9)	37 (3.7)	22 (4.3)		
**Creatinine, μmol/L**	63.9 (54.5-75.8)	62.9 (54.3-73.68)	66.4 (55.05-78.6)	<0.001*	64.0-104.0
64.0-104.0	684 (44.9)	440 (43.7)	244 (47.3)	0.001*	
< 64.0	756 (49.6)	526 (52.2)	230 (44.6)		
> 104.0	84 (5.5)	42 (4.2)	42 (8.1)		
**Interleukin-6, pg/mL**	1.5 (1.5-4.13)	1.5 (1.5-3.75)	1.5 (1.5-4.67)	0.532	0-7.0
0-7.0	562 (83.1)	333 (84.5)	229 (81.2)	0.257	
>7.0	114 (16.9)	61 (15.5)	53 (18.8)		
**SARS-CoV-19 IgM**				0.391	-
NO	343 (65.3)	149 (67.4)	194 (63.8)		
YES	182 (34.7)	72 (32.6)	110 (36.2)		
**SARS-CoV-19 IgG**				0.164	-
NO	46 (9.3)	15 (7.1)	31 (10.8)		
YES	451 (90.7)	195 (92.9)	256 (89.2)		

Note: * represents *p* value <0.05.

**Table 3:** Blood coagulation test results of 1525 patients with COVID-19

Covariate	All patients (n=1525) Median (IQR) / n (%)	Normal PCT (n=1008) Median (IQR) / n (%)	Elevated PCT (n=517) Median (IQR) / n (%)	*P* value	Reference range
**Prothrombin time (s)**	11.3 (10.8-11.7)	11.25 (10.7-11.7)	11.3 (10.9-11.7)	<0.001*	9.4-12.5
9.4-12.5	1406 (92.2)	927 (92.0)	479 (92.6)	0.315	
< 9.4	1 (0.1)	0 (0)	1 (0.2)		
> 12.5	118 (7.7)	81 (8.0)	37 (7.2)		
**International Normalized Ratio**	0.97 (0.93-1.01)	0.97 (0.92-1.01)	0.97 (0.93-1.01)	<0.001*	0.8-1.3
0.8-1.3	1457 (95.5)	961 (95.3)	496 (95.9)	0.375	
< 0.8	13 (0.9)	7 (0.7)	6 (1.2)		
> 1.3	55 (3.6)	40 (4.0)	15 (2.9)		
**Activated partial thromboplastin time (s)**	26.8 (23.7-30.15)	25.9 (22.8-29.1)	28.5 (25.65-31.45)	<0.001*	25.1-36.5
25.1-36.5	906 (65.0)	526 (59.1)	380 (75.5)	<0.001*	
< 25.1	412 (29.6)	319 (35.8)	93 (18.5)		
> 36.5	75 (5.4)	45 (5.1)	30 (6.0)		
**Fibrinogen, (g/L)**	2.9 (2.34-3.68)	2.9 (2.27-3.68)	2.8 (2.43-3.58)	0.004*	2.38-4.98
2.38-4.98	1162 (76.2)	777 (77.1)	385 (74.5)	0.524	
< 2.38	270 (17.7)	172 (17.1)	98 (19.0)		
> 4.98	93 (6.1)	59 (5.9)	34 (6.6)		
**Thrombin time (s)**	17.4 (16.6-18.2)	17.5 (16.5-18.4)	17.4 (16.7-17.9)	<0.001*	10.3-16.6
10.3-16.6	215 (15.4)	114 (12.8)	101 (20.1)	<0.001*	
> 16.6	1178 (84.6)	776 (87.2)	402 (79.9)		
**D-dimer (g/L)**	0.34 (0.18-0.88)	0.35 (0.17-0.88)	0.33 (0.2-0.88)	0.020*	0-0.5
0-0.5	796 (57.1)	486 (54.6)	310 (61.6)	0.011*	
> 0.5	797 (42.9)	404 (45.4)	193 (38.4)		

Note: * represents *p* value <0.05.

**Table 4:** Clinical treatment and outcomes of 1525 patients with COVID-19

Covariates	All patients (n=1525) n (%)	Normal PCT (n=1008) n (%)	Elevated PCT (n=517) n (%)	*P* value
**Drugs**				
Antibiotic	441 (28.2)	348 (34.5)	93 (18.0)	<0.001*
Antiviral drugs	727 (98.9)	610 (99.7)	117 (95.1)	<0.001*
Antimalarial drugs	113 (71.5)	89 (90.8)	24 (40.0)	<0.001*
Anticoagulants	122 (8.5)	52 (5.2)	70 (13.5)	<0.001*
Corticosteroid	99 (6.5)	72 (7.1)	27 (5.2)	0.150
Vitamin C	219 (100.0)	140 (14.7)	101 (14.6)	-
Traditional Chinese medicine	1298 (100.0)	812 (85.3)	594 (86)	-
**Oxygen Support**				0.854
Low-flow nasal cannula	219 (85.2)	156 (85.7)	63 (84.0)	
Non-invasive ventilation or high-flow nasal cannula	32 (12.5)	22 (12.1)	10 (13.3)	
Invasive mechanical ventilation	5 (1.9)	3 (1.6)	2 (2.7)	
ECMO	1 (0.4)	1 (0.5)	0 (0)	
**CT scores**				
1-4	69 (37.5)	32 (28.3)	37 (52.1)	0.001*
5-7	115 (62.5)	81 (71.7)	34 (47.9)	
**Disease Progression**				<0.001*
Stableness/Hospitalisation	33 (2.2)	10 (1.0)	23 (4.4)	
Improvement/Recover	1479 (98.2)	992 (98.4)	487 (94.2)	
Death	13 (0.9)	6 (0.6)	7 (1.4)	
Days in hospital, median (IQR)	18 (13-24)	21 (15-28)	14 (10-18)	<0.001*
**Severity on admission**				
Mild	537 (34.3)	520 (51.6)	17 (3.3)	<0.001*
General	742 (47.4)	309 (30.7)	392 (75.8)	
Severe	263 (16.8)	174 (17.3)	89 (17.2)	
Critical	24 (1.5)	5 (0.5)	19 (3.7)	
**Severity at worst**				
General/Mild	752 (49.5)	444 (44.2)	308 (59.7)	<0.001*
Severe	723 (47.6)	550 (54.8)	173 (33.5)	
Critical	45 (3.0)	10 (1.0)	35 (6.8)	

Note: * represents *p* value <0.05.

**Table 5:** Cox regression analysis

Cox Regression Analysis	Group	HR		95 % CI	*P* value
Univariate Analysis	Normal PCT	ref			
	Elevated PCT	3.377	1.102	10.344	0.033
Multivariate Analysis^*^	Normal PCT	ref			
	Elevated PCT	4.933	1.170	20.788	0.030

*Adjusted for age, the history of cardiovascular disease, WBC, PLT, lymphocyte count and D-dimer.

**Table 6:** Logistic regression analysis

Logistic Regression Analysis	Group	OR		95 % CI	*P* value
Univariate Analysis	Normal PCT	ref			
	Elevated PCT	7.247	3.559	14.757	<0.001
Multivariate Analysis^*^	Normal PCT	ref			
	Elevated PCT	10.679	4.562	25.000	<0.001

*Adjusted for age, the history of cardiovascular disease, WBC, PLT, lymphocyte count and D-dimer.

## Abbreviations

Coronavirus disease 2019: COVID-19
PCT: Procalcitonin
